# Comparative analysis of antigen coding genes in 15 red cell blood group systems of Yunnan Yi nationality in China: A cross‐sectional study

**DOI:** 10.1002/hsr2.891

**Published:** 2022-10-17

**Authors:** Kun‐Hua He, Lu‐Qiong Xu, Ying‐Feng Hu, Yin‐Xia Xu, Yu Zhao, Jing‐Yan Bao, Bu‐Qiang Wang

**Affiliations:** ^1^ Department of Blood Transfusion Qujing No.1 Hospital of Yunnan Province Qujing China; ^2^ Department of Research and Development Jiangsu LIBO Medicine Biotechnology Co., Ltd Jiangyin China

**Keywords:** A4GALT, genetic polymorphism, P1PK, rare blood type, Yunnan Yi nationality

## Abstract

**Introduction:**

There are few analyses of the 15 red blood group system antigen coding genes found in the Yunnan Yi nationality. This has caused many poteintial dangers relating to clinical blood transfusion. In this report, the coding genes and distribution of 15 blood group antigens system in the Yi nationality were tested and compared with those of Han nationality and other ethnic minorities.

**Methods:**

The samples came from the healthy subjects in the first people's Hospital of Qujing, Yunnan Province. Two hundred and three Yunnan Yi and 197 Han nationality individuals were included. Thirty‐three blood group antigens with a low frequency from the 15 blood group systems of Yunnan Yi blood donors were genotyped and analyzed by PCR‐SSP. Sanger sequencing was used to detect A4GALT from the Yunnan Yi nationality. The *χ* ^2^ test was used to analyze observed and expected values of gene distribution to verify conformation to the Hardy‐Weinberg equilibrium law. Fisher's exact test was used to analyze gene frequency distribution, and the statistical significance was set at *p* < 0.05.

**Results:**

The ABO blood group examination results for the Yi nationality and the local Han nationality in Qujing City, Yunnan Province, showed the majority were type A and type O, while the least prevalent was type AB. RhD+ accounts for more than 98% of the Yi and Han populations. There was a significant difference in ABO blood group antigen distribution between these two nationalities (*p* < 0.05), but there was no significant difference in the composition ratio of D antigen in the Rh blood group system (*p* > 0.05). Compared with Tibetan (Tibet), Zhuang (Nanning), and Dong (Guangxi), the gene distribution frequencies of Rh blood group system phenotype CC were significantly lower in the Yunnan Yi nationality (*p* < 0.05). There were significant differences in six erythrocyte phenotypic antigens in the Yi nationality in Yunnan compared with Han nationality, such as LW(a−b−), JK(a−b+), MMSs, Di(a−b+), Wr(a−b−), and Kp(a−b+) (*p* < 0.05). There were gene phenotypes with a low frequency in the four rare blood group systems: LW, MNS, Wright, and Colton. Several different mutation types occurred in the P1PK blood group system's A4GALT gene.

**Conclusion:**

Yunnan Yi nationality has a unique genetic background. There are some significantly different distributions of blood group system genes with a low frequency in different regions and groups in China. Multiple mutations in the A4GALT gene of the P1PK blood group system may be related to their environment and ethnic evolution.

## 1 INTRODUCTION

The red cell blood group systems is of great importance to clinical transfusion medicine, and until now 43 have been identified by International Transfusion Association (ISBT).^1^ The genetic background of the erythroid blood group system is polymorphism, and the gene frequency distribution is related to ethnicity and region.^2^ Erythroblood group system antibodies cause fetal and neonatal hemolytic disease (hemolytic disease of the fetus and newborn, HDFN) and hemolytic transfusion response (hemolytic transfusion reactions, HTRs).

The ABO blood group system is one of the most important human erythroid blood group systems.^3^ It consists of four antigens (A, B, A1 and A, B). These antigens, called oligosaccharide antigens, are widely expressed in erythrocyte membranes, tissue cell membranes, and in saliva and humoral fluids.^4^ It is important for the diagnosis and treatment of cross‐mating, neonatal hemolysis, and organ transplantation. The Yi of Yunnan province have a unique genetic background, the antigen distribution of its blood group system with a low frequency has not until now been fully reported, and there is no large sample of gene polymorphism in Yunnan Yi population. We found two cases of P phenotype in a previous study, and gene sequencing found a new A4GALT allele c.456_457_insACACCCC homozygous mutation (NCBI number: MG812384), which is the molecular formation mechanism of the p phenotype.^5^ A4GALT polymorphisms and rare Au(a−b+) individuals were also found in this pedigree. Therefore, it is speculated that A4GALT and blood type antigen genes with a low frequency have multiple polymorphisms in the Yunnan Yi population.

It was reported that the antigen of the P1PK blood group system was not confirmed by the ISBT until 2011. The system has three antigens, respectively: P1, P^k^, and NOR.^6^ The locus of the P1PK antigen is sub‐band 2 in region 1 of the long arm of chromosome 22,22q.^7^, ^8^ This gene is called A4GALT. The gene product is: 4‐ ‐galactosyltransferase, consisting of 353 amino acids. The A4GALT gene is polymorphic with 52 alleles, with most gene mutations occurring on exon 3.^9^, ^10^, ^11^ The A4GALT locus encodes a glycosyltransferase that synthesizes the terminal galactose α1‐4Gal of P^K^ (Gb3/CD77) glycosphingolipid α1‐4Gal, which plays an important role in transfusion medicine, obstetrics and pathogen susceptibility.^12^ Anti‐P1 antibodies is associated with a hemolytic transfusion response, whereas P and PK‐related antibodies are associated with hemolytic transfusion response, neonatal hemolytic disease, and spontaneous abortion.^13^, ^14^ In addition to the P1Pk blood group system, whether the distribution of other blood group systems is unique in the Yunnan Yi population deserves our study. For example, the Rh blood group system contains two genes, RhD and RhCE, and the expressed antigen is a 12‐transmembrane glycoprotein. Due to RhD blood group incompatibility, neonatal hemolysis will occur.^15^, ^16^

The Yi nationality is the largest ethnic minority in Yunnan, China, with a unique genetic background due to ethnic migration and integration. In this study, PCR‐SSP genotype analysis of P1PK blood type A4GALT, and the study of distribution characteristics in their gene polymorphisms, provide a data basis and assist in the establishment of a comprehensive rare blood type database.

## 2 METERIALS AND METHODS

### 2.1 Blood samples

Samples of three generations of unrelated Han and Yi people in Fuyuan County, Qujing City, Yunnan Province, China, were randomly collected from December 9 to December 12th, 2018, with a total of 400 blood samples from healthy people, including 203 Yi, 197 Han, 203 men, and 197 women. Healthy population EDTA anticoagulant samples were 8 ml, centrifuged at 1500 g for 5 min, and patient serum (plasma) were isolated and stored at 4°C. This experiment was subject to informed consent from the hospital ethics committee and patients, ethics number is IRB2018‐001(S)‐01.

### 2.2 Extraction of genomic DNA

Genomic DNA, at an OD_260/280_ ratio was extracted from whole blood between 1.8 and 2.0 using the TIAN amp Blood DNA Kit (Jiangsu Zhongji Wantai Biomedicine, cat: 20190215) and kept for standby at −20°C.

### 2.3 PCR amplification

A PCR reaction system was prepared, and bidirectional primers were added at a concentration of 10  pmol/L (Supporting Information: Table S1 for the primer sequence). The PCR products were subjected to agarose electrophoresis, and the target fragments were cut in a 2.0 ml centrifuge tube and recovered using a glue recovery kit. The recovered products were sequenced.

### 2.4 Genotyping detection

Detect 12 clinically important erythrocyte antigen genes by RT‐qPCR with human erythrocyte rare blood group genotyping kit, add specific primers (Supporting Information: Table S1) to amplify PA and PB genes by PCR, then sequence amplified products by Sanger sequencing, and finally compare the sequencing results to obtain the mutation point (mutation position) and then determine the genotype.

### 2.5 Statistical analysis

Statistical analysis was performed using SPSS 22.0. and Counting data are expressed as percentages. The observed and expected values of the gene distribution were analyzed by *χ* ^2^ test to verify their compliance with the Hardy‐Weinberg equilibrium. Fisher's exact test was used to analyze the gene frequency distributions of both the Yi and Han nationality, and *p* < 0.05 was considered statistically significant. The rare blood type gene frequency distribution between Yi and Han was compared and the polymorphisms of the P1PK blood type in the Yunnan Yi nationality was analyzed.

## 3 RESULTS

### 3.1 Serological examination results of ABO blood group system and RHD antigen

From the collection of 203 Yunnan Yi samples and 197 Han samples, ABO blood type was analyzed (Supporting Information: Table S2): Type A was 35.5% (72/203); type B was 24.1% (49/203); type O was 35.5% (72/203); and type AB was 4.9% (10/203). The *χ* ^2^ test was applied to ABO blood types found between the Yi and Han nationalities, where significant differences were found in ABO blood antigen distribution (*p* = 0.025). The antigen frequency of RhD^+^ was 99.5% (202/203) and RhD^−^ was 0.5% (1/203). Han nationality (197 cases) type A was 31.5% (62/197), type B was 27.4% (54/197), type O was 28.4% (56/197); type AB was 12.7% (25/197); the RhD^+^ antigen frequency was 99.0% (195/197), and RhD^−^ was 0.5% (1/197). The Yi nationality had mostly A and O blood, similar to the Han.

The distribution of Rh blood group system alleles is also significantly different among ethnic minorities in different regions of China (Supporting Information: Table S3). The Yunnan Yi's Rh system CC phenotype frequency showed a significant decrease (*p* < 0.001), when compared with Tibetan (Tibet) and Dong (Guangxi), while EE was significantly increased when compared to the above (*p* < 0.001), with exception of Tibetan, where it was significantly lower (*p* < 0.01). This shows that the Rh phenotypes CC and EE in the Yunnan Yi nationality have a unique genetic background and measurable differences compared with ethnic minorities in other regions.

### 3.2 Detection of blood groups with a low frequency of Yunnan population in China

Rare blood type genotype and gene frequency in the Yunnan Yi nationality (Table 1). Except for LW(a−b−) genotype in LW blood group system (*p* < 0.001), the genotype distribution of blood groups with a low frequency of Yi nationality accorded with the Hardy‐Weinberg equilibrium (*p* > 0.05). The gene frequency of blood groups with a low frequency in the Yi population remained stable. It was found that there are four rare blood group systems and rare phenotypes in the Yunnan Yi nationality: LW(a−b−) = 0.0246 (five cases); MMSS = 0.0049 (one case); MNSS = 0.0049 (one case); MM = 0.0394 (eight cases); MN = 0.0148 (three cases); Wr(a−b−) = 0.0591 (12 cases); Co(a−b−) = 0.0049 (one case).

**Table 1:** Rare blood type genotype and antigen frequency of Yunnan Yi nationality, China (*n*= 203)

Blood‐group system	Genotype	Observed number	Expected value	Allele	Gene frequency	*χ* ^2^	*p*‐value
Rh	Ce/cE	91 (0.4483)	91.86 (0.4525)	Ce	0.4433	2.712	0.91
	ce/ce	3 (0.0148)	3.55 (0.0175)	ce	0.2882		
	CE/Ce	3 (0.0148)	2.54 (0.0125)	cE	0.2611		
	cE/cE	13 (0.0640)	8.63 (0.0425)	CE	0.0074		
	Ce/ce	13 (0.0640)	17.26 (0.0850)				
	cE/ce	7 (0.0345)	8.63 (0.0425)				
	CE/cE	0 (0)	0.50 (0.0025)				
	CE/ce	73 (0.3596)	70.04 (0.3450)				
Lw	Lw(a−b−)	5 (0.0246)	6.9 (0.0340)			26.448	<0.001
	Lw^b^/Lw^b^	2 (0.0099)	0.1 (0.0000)	Lw^a^	0.4557		
	Lw^a^/Lw^a^	194 (0.9557)	192.1 (0.9460)	Lw^b^	0.0148		
	Lw^a^/Lw^b^	2 (0.0099)	3.9 (0.0190)				
Duffy	Fy^a^/Fy^a^	181 (0.8916)	178.13 (0.8775)	Fy^a^	0.9458	/	0.76
	Fy^a^/Fy^b^	22 (0.1084)	24.87 (0.1225)	Fy^b^	0.0542		
Kidd	Jk^b^/Jk^b^	54 (0.2660)	43.14 (0.2125)			1.79	0.41
	Jk^a^/Jk^a^	43 (0.2118)	42.63 (0.2100)	Jk^a^	0.4729		
	Jk^a^/Jk^b^	106 (0.5222)	117.23 (0.5775)	Jk^b^	0.5271		
MNSs	MMss	70 (0.3448)	63.95 (0.3150)			6.839	0.74
	NNss	22 (0.1084)	30.96 (0.1525)	M	0.6576		
	MNss	83 (0.4089)	82.22 (0.4050)	N	0.3424		
	MMSs	8 (0.0394)	6.09 (0.0300)	S	0.0443		
	NNSs	1 (0.0049)	0.51 (0.0025)	s	0.8990		
	MNSs	6 (0.0296)	7.61 (0.0375)	Mur	0.0172		
	MMSS	1 (0.0049)	0.51 (0.0025)				
	MNSS	1 (0.0049)	0.51 (0.0025)				
	MM–	8 (0.0394)	5.58 (0.0275)				
	NN–	0 (0)	3.55 (0.0175)				
	MN–	3 (0.0148)	1.52 (0.0075)				
Miltenberg	Mur‐	198 (0.9754)	196.91 (0.9700)			/	1.00
	Mur+	5 (0.0246)	6.09 (0.0300)				
Scianna	Sc1/Sc1	203 (1.0000)	203 (1.0000)	Sc1	1.0000	/	1.00
	Sc2/Sc2	0 (0)	0 (0)	Sc 2	0		
Diego	Di^b^/Di^b^	186 (0.9163)	176.10 (0.8675)	Di^a^	0.0410	3.16	0.22
	Di^a^/Di^a^	0 (0)	0.51 (0.0025)	Di^b^	0.9590		
	Di^a^/Di^b^	17 (0.0837)	26.39 (0.1300)				
Wright	Wr(a−b−)	12 (0.0591)	18.27 (0.0900)	Wr^a^	0.0345	3.2	0.36
	Wr^b^/Wr^b^	179 (0.8818)	178.64 (0.8800)	Wr^b^	0.9064		
	Wr^a^/Wr^a^	2 (0.0099)	1.02 (0.0050)				
	Wr^a^/Wr^b^	10 (0.0493)	5.08 (0.0250)				
Kell	k/k	199 (0.9803)	199.45 (0.9825)	K	0.0099	/	1.00
	K/k	4 (0.0197)	3.55 (0.0175)	k	0.9901		
	Kp^b^/Kp^b^	190 (0.9360)	194.37 (0.9575)	Kp^a^	0.0320	/	0.51
	Kp^a^/Kp^b^	13 (0.0640)	8.63 (0.0425)	Kp^b^	0.9680		
Colton	Co(a−b−)	1 (0.0049)	0.50 (0.0025)			0.208	0.90
	Co^a^/Co^a^	199 (0.9803)	201 (0.9901)	Co^a^	0.9877		
	Co^a^/Co^b^	3 (0.0148)	1.50 (0.0074)	Co^b^	0.0074		
Domocrock	Do^b^/Do^b^	171 (0.8424)	170.52 (0.8400)	Do^a^	0.0788	/	1.00
	Do^a^/Do^b^	32 (0.1576)	32.48 (0.1600)	Do^b^	0.9212		
Lutheran	Lu^b^/Lu^b^	149 (0.7340)	151.74 (0.7475)	Lu^a^	0.1330	/	0.82
	Lu^a^/Lu^b^	54 (0.2660)	51.26 (0.2525)	Lu^b^	0.8670		
Yt	Yt^a^/Yt^a^	203 (1.0000)	203 (1.0000)	Yt^a^	1.0000	/	1.00
	Yt^b^/Yt^b^	0 (0)	0 (0)	Yt^b^	0		

### 3.3 Comparison of phenotypic frequency distribution of rare blood group system between Yunnan Yi and Han

After Fisher exact verification, compared with the local Han nationality: Lw(a−b−), Jk(a−b+), MMss, Di(a−b+), Wr(a−b−), and Kp(a−b+) had significant differences (*p* < 0.05) (Table 2).

**Table 2:** Comparison of phenotype frequency distribution of Yunnan Yi and Han nationality

Blood group system	Phenotype	Han	Yi	*χ* ^2^	*p*‐value
RH	Ccee	0.4569	0.4483	8.901	0.26
	ccee	0.0203	0.0148		
	CCEe	0.0102	0.0148		
	ccEE	0.0203	0.0640		
	Ccee	0.1066	0.0640		
	ccEe	0.0508	0.0345		
	CcEE	0.0051	0.0000		
	CcEe	0.3299	0.3596		
LW	Lw(a–b–)	0.3350	0.0246	68.546	<0.001
	Lw(a–b+)	0.0000	0.0099		
	Lw(a+b–)	0.6650	0.9557		
	Lw(a+b+)	0.0000	0.0099		
Duffy	Fy(a+b‐)	0.8629	0.8916	/	0.446
	Fy(a+b+)	0.1371	0.1084		
Kidd	Jk(a‐b+)	0.1574	0.2660	7.746	0.021
	Jk(a+b–)	0.2081	0.2118		
	Jk(a+b+)	0.6345	0.5222		
MNSs	MMss	0.2843	0.3448	23.513	0.009
	NNss	0.1980	0.1084		
	MNss	0.4010	0.4089		
	MMSs	0.0203	0.0394		
	NNSs	0.0000	0.0049		
	MNSs	0.0457	0.0296		
	MMSS	0.0000	0.0049		
	MNSS	0.0000	0.0049		
	MM–	0.0152	0.0394		
	NN–	0.0355	0.0000		
	MN–	0.0000	0.0148		
Miltenberg	Mur‐	0.9645	0.9754	/	0.57
	Mur+	0.0355	0.0246		
Scianna	Sc1+2‐	1.0000	1.0000		1.00
	Sc1–2+	0.0000	0.0000		
Diego	Di(a–b+)	0.8173	0.9163	8.944	0.011
	Di(a+b–)	0.0051	0.0000		
	Di(a+b+)	0.1777	0.0837		
Wright	Wr(a–b–)	0.1218	0.0591	16.01	0.001
	Wr(a–b+)	0.8782	0.8818		
	Wr(a+b–)	0.0000	0.0099		
	Wr(a+b+)	0.0000	0.0493	/	
Kell	kk	0.9848	0.9803		1
	Kk	0.0152	0.0197	/	
	Kp(a–b+)	0.9797	0.9360		0.045
	Kp(a+b+)	0.0203	0.0640	3.921	
Colton	Co(a–b–)	0.0000	0.0049		0.141
	Co(a+b–)	1.0000	0.9803		
	Co(a+b+)	0.0000	0.0148		
Domocrock	Do(a–b+)	0.8376	0.8424	/	1.00
	Do(a+b+)	0.1624	0.1576		
Lutheran	Lu(a–b+)	0.7614	0.7340	/	0.566
	Lu(a+b+)	0.2386	0.2660		
Yt	Yt(a+b–)	1.0000	1.0000	/	1.00
	Yt(a–b+)	0.0000	0.0000		

### 3.4 Rare blood group phenotype distribution differences in the Yunnan Yi nationality andother ethnic minorities in different regions of China

There was significant difference between Yunnan Yi nationality and other ethnic minorities (*p* < 0.001). Compared with other ethnic minorities, the Yi nationality in Yunnan has significant difference (*p* < 0.05). (Table 3).

**Table 3:** Distribution of rare blood type system phenotypes among ethnic minorities in different regions of China

Phenotype	Yunnan Yi nationality	Sichuan Yi nationality^2^	Tibat Tibetan nationality^17^	Xinjiang Kazakhstan nationality^18^, ^19^	Xinjiang Kirkgiz nationality^20^	Harbin Man nationality^21^
	(*n* = 203)	(*n* = 120)	(*n* = 409)	(*n* = 196)	(*n* = 113)	(*n* = 200)
Fy(a+b–)^a^	0.8916	0.8917	0.8484	0.5741	0.5841	0.8950
Fy(a+b+)	0.1084	0.1083	0.1467	0.3672	0.3894	0.1050
Fy(a–b+)	0.0000	0.0000	0.0049	0.0587	0.0265	0.0000
Jk(a+b–)^a^	0.2118	0.3083	0.2910	0.2902	0.3451	0.3800
Jk(a+b+)	0.5222	0.4333	0.5208	0.5008	0.5398	0.3700
Jk(a–b+)	0.2660	0.2583	0.1883	0.2089	0.1150	0.2500
MM	0.4286	0.4667	0.4474	0.4179	/	/
MN	0.4581	0.4333	0.4670	0.4592	/	/
NN	0.1133	0.1000	0.0856	0.1229	/	/
SS^b^	0.0099	0.0083	0.0171	0.0122	/	0.0000
Ss	0.0739	0.1667	0.2592	0.4153	/	0.1250
ss	0.8621	0.8250	0.7237	0.5724	/	0.8750
Di(a–b+)^a^	0.9163	0.9667	0.9340	0.6428	0.8938	0.9443
Di(a+b+)	0.0837	0.0333	0.0636	0.3563	0.0973	0.0549
Di(a+b–)	0.0000	0.0000	0.0024	0.0009	0.0088	0.0008
kk	0.9803	/	1.0000	0.9766	0.9823	/
Kk	0.0197	/	/	0.0234	0.0177	/
Co(a–b–)^a^	0.0049	0.0000	0.0000	/	0.0000	/
Co(a+b–)	0.9803	1.0000	0.9951	/	1.0000	/
Co(a+b+)	0.0148	0.0000	0.0049	/	0.0000	/
Do(a–b+)^a^	0.8424	0.7583	0.7115	0.6075	0.5841	0.7500
Do(a+b+)	0.1576	0.2333	0.2763	0.3465	0.3540	0.2500
Do(a+b–)	0.0000	0.0083	0.0122	0.0461	0.0619	0.0000
Lu(a–b+)^a^	0.7340	1.0000	1.0000	/	/	/
Lu(a+b+)	0.2660	0.0000	0.0000	/	/	/

^a^ There was significant difference between Yunnan Yi nationality and other ethnic minorities (*p*< 0.001).

^b^ Compared with other ethnic minorities, the Yi nationality in Yunnan has significant difference (*p*< 0.05).

## 4 DISCUSSION

In this study, results indicated that the Yi and local Han people in Qujing City, Yunnan Province are mostly A‐type and O‐type. There was a significant difference in the antigen distribution of ABO blood group system between the two ethnic groups (*p* < 0.05), while the D antigen composition ratio of Rh blood group system was not significant (*p* > 0.05). Compared with Tibetan Tibetan, Nanning Zhuang, and Guangxi Dong, the distribution frequencies of CC and EE genes in the Rh blood group system were significantly lower in Yunnan Yi (*p* < 0.05). There were significant differences between the six antigens of Yunnan Yi people, including Lw(a−b−), Jk(a−b+), MMss, Di(a−b+), Wr(a−b−), Kp(a−b+) (*p* < 0.05). There are rare genotypes in the four rare blood group systems of LW, MNS, Wright and Colton. Due to the limited data collected, it may not fully represent the blood group status of the Yi people in Yunnan.

The erythroid blood group system is important for clinical transfusions among populations with high genetic polymorphisms and when there are differences between ethnic groups and regions.^17^ At present, clinical blood transfusion mainly focuses on ABO and Rh blood group systems,^18^ but pay less attention to other blood group systems with a low frequency such as Duffy and MNS. In addition, patients with multiple clinical blood transfusions are often accompanied by the emergence of cross‐blood mismatch or irregular antibodies, causing incompatibility difficulties, which can endanger the lives of patients. Therefore, understanding the characteristics of gene frequency distribution of the many erythroid blood group systems in a region is of great significance to guiding clinical and rational blood use safety, especially in patients who need repeated blood transfusions.

It is well known that the ABO (ISBT, 001) and Rh (ISBT, 004) blood group systems are the two most important systems in the human erythroid blood group system, both of which are autosomal dominant, according to Mendel's Law.^19^, ^20^ Here, we investigated the distribution and gene frequency of 15 blood group system antigens in the Yi and Han people in Yunnan, China, by collecting 203 samples from the Chinese Yunnan Yi nationality and 197 from the Han nationality, and examining their ABO blood type. It was found that type A blood from the Yunnan Yi nationality accounted for the highest proportion, followed by type B and type O. RhD^+^ accounted for 99.51%. The distribution of ABO in the Han nationality showed A to be the most frequent type, reaching 31.47%, followed by O, accounting for 28.43%, while RhD^+^ was 98.98%.

Through the hardy‐Weinberg fit test, the observed and expected values of the distribution of 13 erythrocyte blood type genotypes in 203 Yunnan Yi people were basically in line with the hardy‐Weinberg genetic balance rule, but the LW blood group system had genetic changes, and the difference was statistically significant (*p* < 0.001). this may be related to the small number of individual samples collected in the clinic, resulting in certain deviations in the statistics.^21^ The study of 15 erythrocyte rare blood group system antigen gene poly morphisms in Yunnan population not only provides date for human population genetics, ethnic migration and blood transfusion treatment of blood group patients, but also improves the construction of Yunnan rare blood group gene database, help to solve the problem of difficult blood type identification and cross‐matching incompltibility, reduce blood transfusion reactions such as immune hemolysis, and provide a strong guarantee for clinical safe blood transfusion and precise blood transfusion therapy.^22^

The results of this study suggest that in the LW blood group system, Lw(a−b+) and Lw(a+b+) blood groups were not detected in the Han population, while in the Yunnan Yi people, the Lw(a−b+) blood group accounted for 0.0099, the proportion of Lw(a+b+) blood group is 0.0099, which is statistically different from the distribution of the Han population in the LW blood group system.^23^, ^24^ In the 203 yunnan Yi people, the proportion of the MMss blood type population reached 0.3448, which was significantly higher than the proportion of the Han population of 0.2843. In terms of the NNss blood type, the proportion of the Yunnan Yi population was significantly reduced. In the MNS blood group system, it can be seen that the differences between different blood types are statistically significant.^25^ In the Diego blood group system, it can be seen that the Di(a−b+) blood type is significantly increased in the Yunnan Yi population compared with the Han population, while the Di(a+b+) blood type is less in the Yunnan Yi population, with *p* < 0.05, which is statistically significant. In the Wright blood group system, the expression of Wr(a+b−) and Wr(a+b+) blood groups were not detected in the Han population, while the frequencies of 0.0099 and 0.0493 were found in the Yunnan Yi population, respectively. The expression difference between the two populations has statistical significance.^26^, ^27^

In this study, the distribution of blood group system genotypes with a low frequency in Yunnan was found significantly different among ethnic minorities in different regions of China.^28^

Subsequently, with the polymorphism of P antigen A4GALT gene polymorphism of P1PK was detected. P1PK blood group system antigen which has a common molecular basis, is encoded by the A4GALT gene.^29^ Therefore, detecting A4GALT gene polymorphism is an effective method to understand the antigen distribution of P1PK in the Yi group. It has been reported that Yunnan Province is a region with a high incidence of thalassemia, and ethnic minorities have a higher carrying rate.^30^ This means that the unique genetic background of ethnic minorities in Yunnan can easily lead to the occurrence of hematological diseases. In previous studies of two rare P phenotypes in Yi families,^5^ it was found that they lack all antigens from the P1PK blood group system. Anti‐P1PK (anti‐Tja) antibody was present in all individuals with the P phenotype, which coagulates all phenotype red blood cells except the P phenotype, resulting in habitual abortion in women early in pregnancy.^31^, ^32^ The A4GALT gene determines the formation of the P phenotype, and its exon 3 variation which causes amino acid changes, may cause inactivation of 1,4‐galactotransferase, resulting in the loss of P1, PK and its downstream P, LKE antigen to form the p phenotype.^33^ In this study, homozygous and heterozygous mutations occurred in the A4GALT gene at 903C > G, and the 903C > G mutation in exon 3 of the A4GALT gene affected P phenotype formation.^34^ Thus leading to HDFN, HTRs and habitual abortion in the Yi nationality. The remaining mutations include homozygous mutations 109A > G, 987G > A; heterozygous mutation sites>G, 903C > G, 987G > G, 987G > A, 100G > A; heterozygous new mutations 493C > T, 892C > A, 463_464insACACCCC, 352C > A, 353C > A, 892C > A//109, 903, 987, are not reported in the literature, and in a future study we will explore the factors causing the p phenotype of the P1PK blood group system.

## 5 CONCLUSION

The Yunnan Yi nationality has a unique genetic background. There are some significantly different distributions of rare blood group system genes in different regions and groups in China. Multiple mutations in the A4GALT gene of the P1PK blood group system may be related to their environment and ethnic evolution.

## AUTHOR CONTRIBUTIONS

**Kun‐Hua He**: Conceptualization; methodology; project administration; supervision; writing–original draft; writing–review and editing. **Lu‐Qiong Xu**: Investigation; validation; visualization. **Ying‐Feng Hu**: Investigation; validation; visualization. **Yin‐Xia Xu**: Investigation; validation; visualization. **Yu Zhao**: Data curation; formal analysis; software. **Jing‐Yan Bao**: Investigation; validation; visualization. **Bu‐Qiang Wang**: Investigation; validation; visualization.

## CONFLICTS OF INTEREST

The authors have no conflicts of interest to declare. All authors have read and approved the final version of the manuscript. They had full access to all of the data in this study and takes complete responsibility for the integrity of the data and the accuracy of the data analysis. Kun‐hua He affirms that this manuscript is an honest, accurate, and transparent account of the study being reported; No important aspects of the study have been omitted; Any discrepancies from the study as planned have been explained.

## ETHICS STATEMENT

This study was approved by the Medical Ethical Committee of Qujing No.1 Hospital of Yunnan Province, and the ethics number is IRB2018‐001(S)‐01.

## TRANSPARENCY STATEMENT

The lead author Kun‐Hua He affirms that this manuscript is an honest, accurate, and transparent account of the study being reported; that no important aspects of the study have been omitted; and that any discrepancies from the study as planned (and, if relevant, registered) have been explained.

## Supporting information

Supporting information.

## Data Availability

The authors confirm that the data supporting the findings of this study are available within the article and its supplementary materials. The data that support the findings of this study are available on request from the corresponding author. The data are not publicly available due to privacy or ethical restrictions.
