# Supplementary material for: Comparative analysis of antigen coding genes in 15 red cell blood group systems of Yunnan Yi nationality in China: A cross‐sectional study
Source: Health Sci Rep. 2022 Oct 17;5(6):e891. doi: 10.1002/hsr2.891 (PMC9576115; doi:10.1002/hsr2.891)
Supplement: Supplementary file 1 — Supporting information. [file HSR2-5-e891-s001.docx]

**Supplement tables：**

**Table S1 Primer sequences of P1PK and rare blood group genotyping**

| Name | Primer sequences |
| --- | --- |
| PAE3F | GGTGCTCATCTCTTGCAGAC |
| PAE3R | TCTTGTCCCTTCTTCCCCAT |
| PBF | TACCCATCTCTTTCTGTCCC |
| PBR | CACTGACCTCCCCATGAATT |
| RhCF | CTGCTTCCAGTGTTAGGGCG |
| RhCR | GCTTCCGTGTTAACTCCATAGAC |
| RhcF | GTGATGACCACCTTCCCAGG |
| RhcR | ATCTCCCCACCGAGCAGTC |
| RhEF | ACTGTGACCACCCAGCATTCTT |
| RhER | TTCTGGCCAAGTGTCAACTCTC |
| RheF | CCAGGTGGTGGAGGTTGCA |
| RheR | GGATTGGACTTCTCAGCAGAGC |
| LwAF | TTCCCTCTGTCGCTGCTG |
| LwAR | CCCTCACGTCGAGCAGCT |
| LwBF | TTCCCTCTGTCGCTGCTG |
| LwBR | CCTCACGTCGAGCAGCC |
| FaF | GCTGCTTCCAGGTTGGCAC |
| FaR | TCTTCCGGTGTAACTCTGATGG |
| FbF | GCTGCTTCCAGGTTGGCAT |
| FbR | TCTTCCGGTGTAACTCTGATGG |
| JaF | CCCAGAGTCCAAAGTAGATGTC |
| JaR | CCTCCCCACTCATGTGCC |
| JbF | CCCAGAGTCCAAAGTAGATGTT |
| JbR | CCTCCCCACTCATGTGCC |
| MurF | GGTTTCCTCTTCTGGAGGGT |
| MurR | AGACCTGGGCTCAGCTCTATC |
| MF | ATATCAGCATCAAGTACCACTGGT |
| MR | CAGTGACAGGTCCCCTAAAATG |
| NF | TTGAAGTGTGCATTGCCACCT |
| NR | GATGAGAAAACCAAGGCACG |
| SF | AACGATGGACAAGTTGTCCCA |
| SSR | ATCCATGAATACGTGTTGGGTC |
| ssF | ACGATGGACAAGTTGTCCCG |
| ssR | ATCCATGAATACGTGTTGGGTC |
| ScF | CCTCCTTGGGTACCGTCCC |
| ScR | CCTCCTTGGGTACCGTCCT |
| Sc12 | ATCCATGAATACGTGTTGGGTC |
| DiaF | CAGGGCCAGGGAGGCCA |
| DiaR | ACCGCATCTTGCTTCTGTTCA |
| DibF | CAGGGCCAGGGAGGCCG |
| DibR | ACCGCATCTTGCTTCTGTTCA |
| WraF | TGGGCTTGCGTTCCAAGTTT |
| WraR | CCCCTGGCTTTTCACTATTC |
| WrbF | TGGGCTTGCGTTCCGAGTTT |
| WrbR | TGTCTCTCACGTGGTGATCT |
| KF | ACTCATCAGAAGTCTCAGCA |
| KR | CTAGAGGGTGGGTCTTCTTCC |
| kkF | CTGACTCATCAGAAGTCTCAGCG |
| kkR | GACCTTGGGAGAAGGCAGATAA |
| KpaF | CCTCAGAAACTGGAACAGCCA |
| KpaR | CTCTTGTGCCCAGATAGACCA |
| KpbF | CTCAGAAACTGGAACAGCCG |
| KpbR | CTCTTGTGCCCAGATAGACCA |
| CoaF | TTCACGTTGTCCTGGACCG |
| CoaR | CTCTCAGAGGGAATTGAGCACC |
| CobF | TTCACGTTGTCCTGGACCA |
| CobR | CTCTCAGAGGGAATTGAGCACC |
| DoaF | TTGACCTCAACTGCAACCAGTT |
| DoaR | CCTCCTGAAAGAAGAGGCACA |
| DobF | TTGACCTCAACTGCAACCAGTC |
| DobR | CCTCCTGAAAGAAGAGGCACA |
| AuaF | CACCTCAGTCACTCACCGGC |
| AuaR | ATAGCCCGCAGAGCCAATC |
| AubF | CACCTCAGTCACTCACCGGT |
| AubR | ATAGCCCGCAGAGCCAATC |
| YtaF | CATCAACGCGGGAGACTTCC |
| YtaR | CAGATGGACAGACAAAGAGCC |
| YtbF | CATCAACGCGGGAGACTTCA |
| YtbR | CAGATGGACAGACAAAGAGCC |

**Table S2 The detection of ABO blood type system and RhD antigen**

| Blood type | Yi (n=203) | | Han (n=197) | | χ^2^ | P Value |
| --- | --- | --- | --- | --- | --- | --- |
|  | Case number | proportion | Case number | proportion |  |  |
| A | 72 | 35.47% | 62 | 31.47% | 9.330 | 0.025 |
| B | 49 | 24.14% | 54 | 27.41% |  |  |
| O | 72 | 35.47% | 56 | 28.43% |  |  |
| AB | 10 | 4.93% | 25 | 12.69% |  |  |
|  |  |  |  |  | Fisher test | |
| RhD+ | 202 | 99.51% | 195 | 98.98% |  | 1.000 |
| RhD- | 1 | 0.49% | 1 | 0.50% |  |  |

**Table S3 Phenotype distribution of Rh blood group system in different regions of China**

| Phenotype | Case number | Distribution frequency | | | | |
| --- | --- | --- | --- | --- | --- | --- |
|  |  | CC* | Cc | cc | EE# | E |
| Yunnan Yi | 203 | 0.0148 | 0.8719 | 0.1133 | 0.0640 | 0.4089 |
| Tibet Tibetan **^[15]^** | 409 | 0.3280 | 0.4910 | 0.1810 | 0.1174 | 0.4963 |
| Nanning Zhuang**^[16]^** | 2052 | 0.6950 | 0.2710 | 0.3360 | 0.0531 | 0.2373 |
| Guangxi Dong**^[17]^** | 1927 | 0.6580 | 0.3070 | 0.0346 | 0.0178 | 0.2311 |

*Yunnan Yi are compared with Tibetan nationality of Tibet, Zhuang nationality of Nanning and Dong nationality of Guangxi, χ^2^ were 95.028, 789.042 and 246.8 respectively, *P*<0.001 #Yunnan Yi are compared with the Tibetan nationality of Tibet, Zhuang nationality of Nanning and Dong nationality of Guangxi,,χ^2^ are 12.3, 49.71 and 54.03 respectively, *P* =0.002, < 0.001 and < 0.001 respectively.

**Table S4 Detection of** **A4GALT mutation type of P1PK blood type system in Yunnan Yi nationality (A4GALT*P1.01)**

| Mutation type | Discontinuity | Gender | |
| --- | --- | --- | --- |
|  |  | Male | Female |
| Homozygous mutations | 903C>G | 5 | 7 |
|  | 109A>G | 2 | 0 |
|  | 987G>A | 2 | 0 |
| heterozygous mutation | 109A>G | 21 | 20 |
|  | 903C>G | 35 | 25 |
|  | 987G>A | 20 | 19 |
|  | 100G>A | 3 | 5 |
| Heterozygous new mutations | 493C>T | 0 | 1 |
|  | 892C>A | 3 | 1 |
|  | 463_464insACACCCC | 1 | 1 |
|  | 352C>A | 0 | 1 |
|  | 353C>A | 0 | 1 |
|  | 892C>A//109,903,987 | 0 | 1 |
| No mutation | / | 37 | 30 |
